# Time to Renovate the Humor Styles Questionnaire? An Item Response Theory Analysis of the HSQ

**DOI:** 10.3390/bs10110173

**Published:** 2020-11-13

**Authors:** Paul J. Silvia, Rebekah M. Rodriguez

**Affiliations:** Department of Psychology, University of North Carolina at Greensboro, Greensboro, NC 27402, USA; rmrodriguez@uncg.edu

**Keywords:** humor styles, HSQ, sense of humor, item response theory, psychometrics

## Abstract

The Humor Styles Questionnaire (HSQ) is one of the most popular self-report scales in humor research. The present research conducted a forward-looking psychometric analysis grounded in Rasch and item response theory models, which have not been applied to the HSQ thus far. Regarding strengths, the analyses found very good evidence for reliability and dimensionality and essentially zero gender-based differential item functioning, indicating no gender bias in the items. Regarding opportunities for future development, the analyses suggested that (1) the seven-point rating scale performs poorly relative to a five-point scale; (2) the affiliative subscale is far too easy to endorse and much easier than the other subscales; (3) the four subscales show problematic variation in their readability and proportion of reverse-scored items; and (4) a handful of items with poor discrimination and high local dependence are easy targets for scale revision. Taken together, the findings suggest that the HSQ, as it nears the two-decade mark, has many strengths but would benefit from light remodeling.

## 1. Introduction

The typical “conference hotel” for a psychology conference will get renovated every 4–6 years [1], which is a much faster cycle than the self-report scales presented in the meeting rooms and poster sessions downstairs. The typical self-report scale in the behavioral sciences can go a decade or longer before revision, if ever, and many popular scales grow increasingly dated and stale until they fall into disuse. In the present research, we examine the Humor Styles Questionnaire (HSQ) [2], one of the most popular self-report tools in humor research over the last 20 years [3]. Because the HSQ is nearing the 20-year mark, now is a good time for a forward-looking psychometric analysis that clarifies the scale’s strengths and pinpoints opportunities for future improvement. Our analysis applies tools from the family of Rasch and item response theory (IRT) models [4,5], which have apparently not been applied to the HSQ before but offer keen insight into the behavior of items and rating scales. We conclude by outlining suggestions for the future development of the HSQ.

### 1.1. The Humor Styles Questionnaire

Developed by Martin et al. [2], the HSQ is among the most prominent self-report scales in the psychology of humor [6,7]. The scale has been translated into dozens of languages [8] and adapted for versions used in the workplace [9] and with children [10,11]. Several meta-analyses and systematic reviews involving the HSQ [12,13,14,15] and nearly 2000 citations to the original publication (as of November, 2020) attest to its importance to modern humor scholars.

The humor styles model grew out of research on humor’s role in coping, health, and well-being [3]. Martin et al. [2] proposed four humor styles that form a 2 × 2 matrix. Two styles—*affiliative* and *self-enhancing*—are adaptive, positive styles. The affiliative humor style involves using humor to reduce conflict, build group cohesion, and amuse and entertain others. The self-enhancing humor style involves taking a light-hearted, distanced perspective on stressful events and using humor to cope constructively with stressors. The other two styles—*aggressive* and *self-defeating*—are maladaptive, negative styles. Aggressive humor involves using and enjoying hostile humor, such as teasing, ridicule, and mockery. Self-defeating humor involves making jokes at one’s own expense and tolerating disparaging humor, often to gain social acceptance; it is distinct from healthy self-deprecating humor. The positive–negative dimension is crossed with a self–other dimension: self-enhancing and self-defeating humor styles are intrapersonal (self-oriented), whereas affiliative and aggressive humor styles are interpersonal (other-oriented).

These four styles are measured with a 32-item self-report scale. Each style is assessed with eight items. Abbreviated versions of the items are shown in Table 1; the full items can be found in the original article (Martin et al. [2], pp. 58, 59). The items are a mix of positively and negatively worded statements, and the respondents indicate their endorsement of each item on a Likert-type scale ranging from 1 (*totally disagree*) to 7 (*totally agree*).

Culture and gender have been major points of interest in research on humor styles. Since the early development of the English HSQ, the scale has been translated into dozens of languages and is a popular tool in cross-cultural studies of humor. A recent project that evaluated humor styles in 28 countries [8] is a sign of the breadth of the scale’s use. Gender, a major variable of interest in humor research more broadly [12,16,17], has been prominent in humor styles research as well. Gender differences in the HSQ were reported in the original article [2] and have been widely reported since. The magnitudes and directions of gender differences vary across studies and countries [8,18], but the most common finding is for men to have relatively higher scores than women.

### 1.2. The Present Research

In the present research, we apply tools from Rasch and item response theory models to gain insight into the behavior of the HSQ. Despite the scale’s wide use and popularity, it has not been examined using these tools. Our aim is to evaluate areas for possible improvement, so we focus on a cluster of core issues: (1) the behavior of the seven-point rating scale versus alternatives; (2) reliability, dimensionality, and local item dependence for the four styles; (3) item difficulty and discrimination, particularly if some subscales are too “easy” or “hard”; (4) test information; (5) possible gender bias (differential item functioning); and (6) item readability and polarity.

## 2. Materials and Methods

### 2.1. Participants

We examined the HSQ with two samples. Our primary sample consisted of 1210 adults—557 women and 652 men, ranging in age from 18 to 80 (*M* = 30.90, *SD* = 11.99, *Mdn* = 27)—who completed the HSQ using a 5-point rating scale. Some of the responses (*n* = 810) come from data collected by open psychometrics and shared in the R package *clustrd* [19]; we collected the remaining responses (*n* = 400) using the Prolific.co survey panel. This 5-point dataset is the primary sample reported throughout the manuscript. Our secondary sample consisted of 532 adults—397 women and 135 men, ranging in age from 18 to 51 (*M* = 19.04, *SD* = 2.39, *Mdn* = 18)—enrolled in psychology courses at the University of North Carolina at Greensboro. This sample completed the HSQ using a 7-point rating scale; this dataset was used to illustrate deficiencies in using 7 response options. In both samples, the responses had been screened for non-native speakers of English (when known) and for careless and inattentive responding using missed directed response items, long-string indices, and Mahalanobis *D* values, conducted with the R package *careless* [20].

### 2.2. Analytic Approach

The data were analyzed in R [21] using *psych* [22] and *TAM* [23]. For the IRT models, we estimated generalized partial credit models (GPCM) in TAM using marginal maximum likelihood. These models estimate difficulty (*b*) and discrimination (*a*) parameters for each item as well as item thresholds (the level of the underlying trait at which people have a 50:50 chance of responding to one or the other scale option). Four models were estimated, one for each HSQ subscale. The data and R code are available at Open Science Framework (Supplementary Materials). For clarity, we combine the results with our explanation of the statistical methods as well as our discussion and interpretation.

## 3. Results and Discussions

### 3.1. Rating Scale Analysis: Is Seven Points Too Many?

The HSQ was developed with a seven-point Likert-type rating scale, a common number in the self-report assessment of individual differences, and most published research appears to have used seven response options. Many researchers, however, have used five-point scales in their HSQ research [24,25], and some have used three-point scales [26]. Some translations of the HSQ have adopted other rating scales, such as the four-point scale used in the Italian version [27]. Notably, the adaptations of the HSQ for children [10,11] use a four-point scale to simplify the judgments for the younger intended population.

The optimal number of rating scale categories—for a particular scale targeted at a particular population—is an empirical question [28]. A common intuition among researchers is that “more is better”: a nine-point scale, for example, will “expand the variance,” as the saying goes, relative to a four-point scale by “giving people more options.” Nevertheless, measurement theory grounded in Rasch and IRT models points out that allowing people to make finer distinctions does not mean that they can make fine distinctions reliably [4]. Researchers can study the empirical behavior of their rating scales to discern the optimal number of categories.

The first step is to evaluate whether the estimated item thresholds are *disordered*. In the GPCM, a seven-point rating scale has six thresholds that represent the “tipping point” between responses. The threshold between 1 and 2 for affiliative humor, for example, represents the trait level of affiliative humor at which someone has a 50:50 chance of responding with a 1 or 2. The thresholds should be monotonically ordered, ascending from smaller (usually negative) values to larger values. When the thresholds are ordered, it means that as the trait increases from low to high, each response option, from lowest to highest, takes its turn as the most likely response. When thresholds are out of order, it is usually a sign that a rating scale has too many categories—the participants are underusing using some of them.

**Figure 1:**
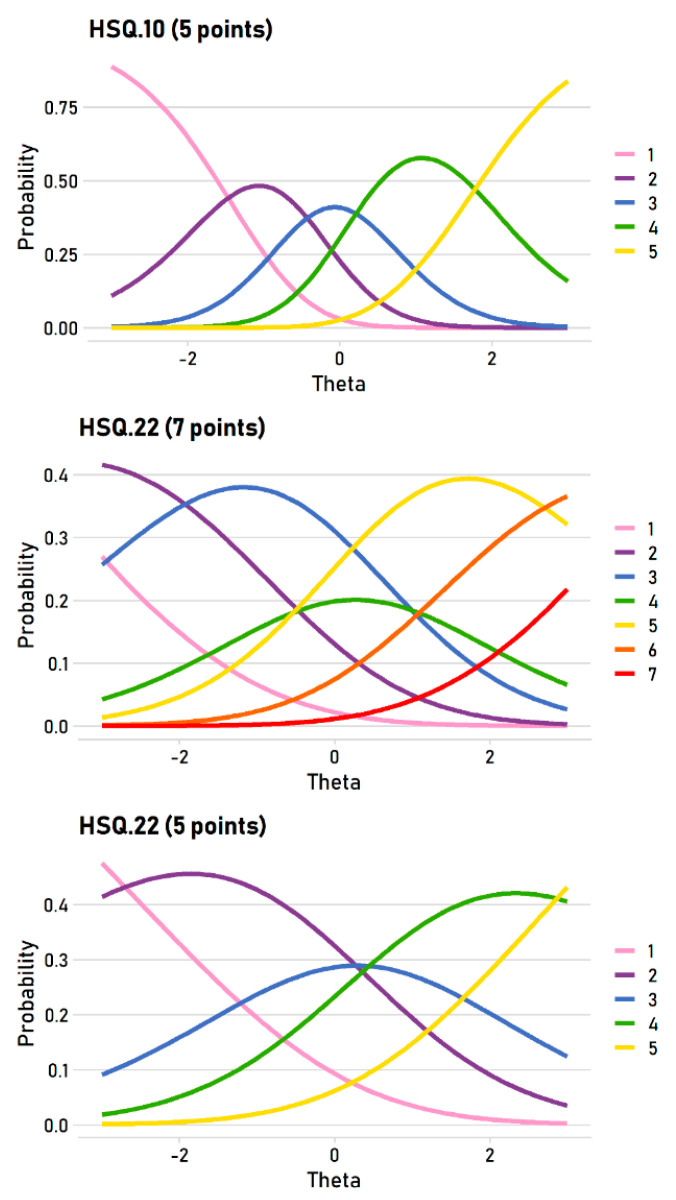
An example of an ideal, ordered pattern of item thresholds (TOP), a disordered item (7-point scale; MIDDLE), and the ordered version of that item (5-point scale; BOTTOM).

This point can be illustrated with item characteristic curves, which display the probability of giving a particular item response at different trait levels. Ideally, each rating scale option will be most probable at some trait level. The top panel in Figure 1 shows the well-behaved, ideal curves using item 10 (self-enhancing: “If I am feeling upset or unhappy I usually try to think of something funny about the situation to make myself feel better”) completed with a five-point scale. As latent self-enhancing scores move along the trait (theta) continuum from low (−3) to high (3), the most probable response shifts from 1 to 5 in ordered steps. Note how each response takes its turn as the most likely responses. All five response options are thus informative.

In the seven-point HSQ data, however, around a third of the items—10 of 32—had disordered thresholds. These appeared in all four subscales: affiliative (items 1 and 25), self-enhancing (items 2 and 22), aggressive (items 3, 7, 15, and 31), and self-defeating (items 12 and 24). As an example of a disordered item, the middle panel of Figure 1 shows the item characteristic curves for item 22 (self-enhancing: “If I am feeling sad or upset, I usually lose my sense of humor”). For this item, the most likely responses do not move in ordered steps: as the trait moves from low to high, the most likely responses are 2, 3, 5, and 6. Three options in the seven-point scale (1, 4, and 7) are never the most likely responses at any trait level, and most of the trait range (from around −2 to +2.5) is covered by only two scale options (3 and 5). It is obvious that seven options is far too many for this item—it is functionally a four-point scale.

The five-point HSQ data, in contrast, had only five disordered items, which are flagged in Table 1. Most disordered items were in the aggressive subscale (items 11, 19, and 27); the remaining subscales had at most one disordered item: affiliative (item 25), self-enhancing (no disordered items), and self-defeating (item 28). The bottom panel of Figure 1 illustrates item 22 again, but for the five-point data. The pattern is ordered because all five options are most likely at some point of the trait. The pattern is not ideal—option 3 is most likely for only a small theta region—but it is much better behaved than the seven-point version.

Taken together, the behavior of the rating scales suggests that seven response options is probably excessive. The participants were not discerning seven levels of the underlying HSQ styles for nearly a third of the items, so their response behavior indicates that they were using fewer categories to classify their level of the respective humor style. For future HSQ development, then, researchers should consider reducing the number of options. Our data show that a five-point scale outperforms a seven-point scale, but we think researchers should explore other rating scales when revising the HSQ, such as the four-point scale used in the Italian HSQ [27] or further examining the three-point scale explored in recent work [26]. The ideal rating scale for an intended respondent population is an empirical issue, so future scale development work should empirically evaluate its rating scale relative to promising alternatives.

### 3.2. Reliability, Dimensionality, and Local Dependence

#### 3.2.1. Reliability

We explored score reliability with a few metrics, shown in Table 2. Cronbach’s alpha was good for all four subscales (α ranged from 0.79 to 0.86). Omega-hierarchical (ω*_H_*; the proportion of total score variance due to a general factor) was good but lower (0.68 to 0.77), suggesting the possible presence of minor factors. Finally, reliability of the expected a posteriori (EAP) trait scores estimated from the GPCM IRT model were good (0.80 to 0.86). Taken together, the present data support the evidence for score reliability found in much past HSQ research [6].

#### 3.2.2. Dimensionality

For the HSQ, we evaluated *essential unidimensionality*, a looser criterion that recognizes that self-report scales for measures of personality traits are rarely strictly unidimensional [29]. We assessed the dimensionality of the HSQ subscales with three methods: parallel analysis [30], Velicer’s [31] minimum average partial (MAP) criterion, and the ratio of the first-to-second eigenvalues (e.g., greater than 4:1) [29]. The factor analyses, conducted in *psych* (Revelle, 2020) using maximum likelihood factor analysis, suggested good evidence for essential (but not strict) dimensionality. Parallel analysis suggested 3 or 4 factors per subscale, but there was clearly a dominant first factor. For all subscales, the MAP and the 4:1 eigenvalue-ratio criteria indicated 1 factor. Taken together, these findings are consistent with past research, which regularly finds good evidence for the factor structure of the HSQ [8].

#### 3.2.3. Local Dependence

Although the HSQ subscales appear essentially unidimensional, the minor secondary factors revealed by the parallel analysis are worth exploring in more detail. In self-report scales, these “nuisance factors” commonly come from a few items that have redundant meanings, overlapping wording, or similar structure (e.g., reverse-scored). Understanding these minor nuisance factors gives useful guidance for future scale revision because they can flag items that are causing deviations from unidimensionality. We thus examined local dependence statistics for the items using the adjusted *Q*3 statistic (a*Q*3), an extension of Yen’s (1984) *Q*3 that corrects for its negative bias by centering the values on the average *Q*3 [32]. As residual correlations, a*Q*3 values are in the *r* metric, and values greater than |0.20| are commonly flagged as violations of local independence [33]. In brief, these residual correlations reveal if two items correlate with each other after accounting for the influence of the common latent factor.

Table 1 notes the items within each HSQ subscale that were flagged for local dependence. These typically reflected “surface dependence” [34], such as overlapping item keywords or items with reversed-scored wording. For the affiliative scale, the two dependent pairs reflected residual correlations between flipped versions of items (e.g., “I laugh and joke a lot with my closest friends” and “I don’t often joke around with my friends”). For the self-enhancing subscale, the largest value appeared (a*Q*3 = 0.39) for a pair of items about finding humor when alone. No local dependence appeared for the aggressive subscale. Finally, for the self-defeating subscale, the cluster of locally dependent items reflected substantive overlap between a group of items about being a target of others’ jokes and laughter.

For future HSQ development, the patterns of local dependence suggest that it would be straightforward to improve the scales’ unidimensionality, such as by omitting or rewording items to reduce surface dependence. For the self-defeating subscale, however, the cluster of items had a shared substantive meaning—being the target of others’ laughter and teasing—which suggests that it may represent a distinct facet of self-defeating humor that could be explored further [35,36].

### 3.3. Item Difficulty, Item Discrimination, and Test Information

The estimates of the generalized partial credit models are shown in Table 1. Item fit was good according to mean-square infit and outfit statistics, for which 1.00 is the ideal value [4]. (Note that *TAM* estimates infit and outfit using individual posterior ability distributions rather than individual ability estimates, so its fit estimates will vary from WLE-based methods [37]). As an additional measure of item fit, we calculated the root mean squared deviation (RMSD) statistic [37,38]. Köhler et al. [37] suggested that RMSD values under 0.02 indicated “negligible” misfit, values between 0.02 and 0.05 reflected “small” misfit, and values between 0.05 and 0.08 reflected “medium” misfit. The RMSD values suggested good item fit: all values were below 0.05 (see Table 1).

#### 3.3.1. Difficulty

**Figure 2:**
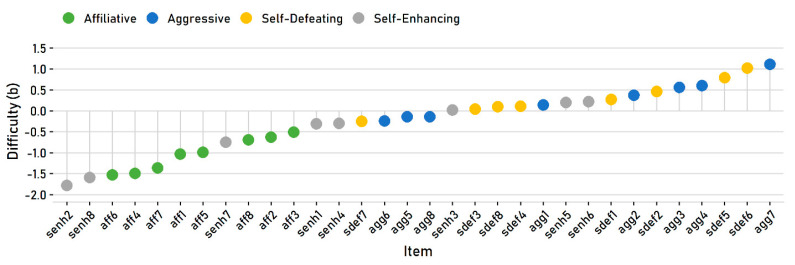
Estimated item difficulty values.

For polytomous self-report items, the item difficulty parameter, known as the *b* parameter, represents the “endorsability” of the item—how easy or how hard it is for people to express agreement with it. The item difficulty values for the HSQ subscales are reported in Table 1. Figure 2 illustrates the difficulty values for all 32 items, which are ordered from “easiest” to “hardest” and color coded by subscale.

The figure highlights a few problematic features of the HSQ. First, the affiliative and self-enhancing subscales are much easier than the others. Most of the self-enhancing items are on the easy end; surprisingly, all eight affiliative items are on the easy end. Most or nearly all of the aggressive and self-defeating items, in contrast, are on the harder end and require relatively high levels of these traits to endorse. The subscales thus vary greatly in their difficulty. For example, the hardest affiliative item is still easier to endorse than even the easiest aggressive and self-defeating items.

#### 3.3.2. Discrimination

**Figure 3:**
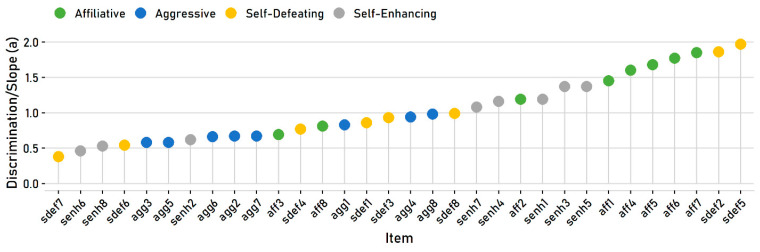
Estimated item discrimination/slope values.

The HSQ subscales also vary in their values on the discrimination *a* parameter (see Table 1), often called the *slope* parameter for polytomous items [39]. In IRT, these values represent how well an item discriminates between people with different trait levels. Items with higher discrimination scores provide more information and can more reliably rank-order participants according to their trait level. They are conceptually equivalent to the magnitude of factor loadings in a confirmatory factor analysis. As Figure 3 illustrates, the HSQ items vary broadly in their discrimination values, from fairly low (below 0.50) to fairly high. Notably, some subscales had more discriminating items. Most of the affiliative items were among the most discriminating items; most of the aggressive items, in contrast, were in the bottom half.

#### 3.3.3. Test Information

**Figure 4:**
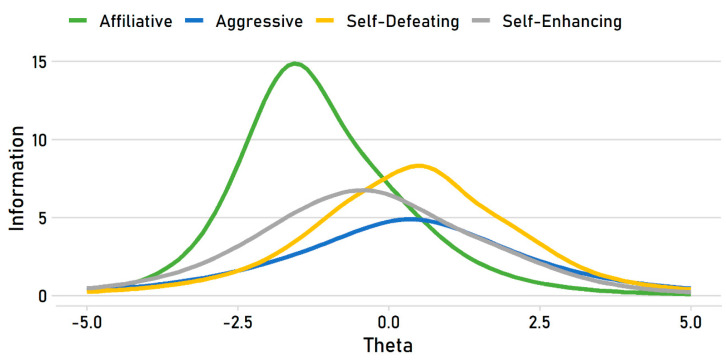
Test information functions for the HSQ subscales.

In item response theory, error is not constant across the trait; some regions of the trait are measured more reliably than others [5]. Test information functions illustrate how much information the subscales provide and at which traits levels they are most reliable. Figure 4 shows the curves for the HSQ subscales, which distill the items’ difficulty and discrimination values into a depiction of how much information they provide across the trait continuum.

The most notable feature is that the affiliative subscale provides much more information relative to the others, but it does so at a much lower trait level. This function reflects the fact that, as we have seen, the affiliative items are very easy but also highly discriminating. The affiliative subscale thus measures the low end of the underlying trait very well—it can make fine distinctions between very low and moderately low levels, for example—but provides much less information about respondents who are high in the affiliative humor style. Stated differently, these items are quite good at rank-ordering people low in affiliative humor but relatively weak at rank-ordering people high in it. The other three subscales have test information functions that are commonly found for measures of individual differences in typical populations: the peaks are in the region of zero, and although they provide less absolute information (i.e., they have lower peaks than the affiliative subscale), they provide information over a much broader range of the trait.

#### 3.3.4. Summary

Of the four HSQ subscales, the affiliative subscale stands out as problematic and worth a close look in future scale development. First, its items are simply too easy for this group of respondents, which represents the typical population for whom the scale is targeted. These items are highly discriminating, which is a strength, but because the items are all fairly easy to endorse, the scale yields a high level of information centered on the low end of the trait. This scale thus does a relatively poor job of sorting people with average and higher levels of the affiliative humor style. Rewording at least some of the items to be harder to endorse would enable the scale to provide more information about high trait levels.

Regarding difficulty and discrimination, the remaining subscales seem solid. A few items with low discrimination levels deserve a look during the item revision process, especially items that were also flagged for disordered thresholds and local dependence.

### 3.4. Gender Bias and Differential Item Functioning

In the present sample, like much past work [2,8], men had at least slightly higher scores on all four subscales. Table 2 reports Cohen’s *d* values and their 95% confidence intervals. For our purposes, the more interesting issue is whether gender differences represent true differences in the underlying trait or if the HSQ items are contaminated with item bias. One of the major virtues of item response analysis is the ability to appraise differential item functioning (DIF). Women and men can vary in their trait scores, of course, but men and women with identical true trait scores should have the same predicted responses. When this condition is violated for an item, it is evidence for DIF. For such items, group differences are difficult to interpret because they are a mixture of true group differences and unwanted item bias.

In the present sample, we evaluated DIF in *lordif* [40,41], which implements a logistic ordinal regression approach using IRT-based trait scores and iterative purification. Because of our large sample size, we used McFadden’s *R*^2^ [42] as an effect size measure of DIF [43]. A criterion of *R*^2^ = 0.01 (more stringent than the common cutoff of *R*^2^ = 0.02 for a small *R*^2^ effect size) was used to flag items for total DIF. Notably, no items were flagged for any subscale by this criterion, indicating essentially zero gender-based DIF for all the HSQ items. This is a striking finding for a self-report scale with widespread gender differences, and it indicates that the gender differences found in the scale scores reflect true group differences rather than item bias.

### 3.5. Readability and Item Polarity

Readability is an important feature of self-report items, but readability statistics are infrequently evaluated for measures of individual differences. For the HSQ, we computed the average words per items and evaluated two common metrics of readability [44] (see Table 2). Higher Flesch “reading ease” scores indicate greater readability, so reading ease is rather easy for the affiliative and self-defeating subscales but fairly hard for the self-enhancing subscale. Gunning’s “fog index” found similar variability between the subscales. The fog index is expressed in American grade levels, so the affiliative subscale had a lower reading level (around 6th grade, 11–12 years old) and the self-enhancing scale had a much higher reading level (around 12th grade, 17–18 years old). The affiliative subscale has mostly short, focused items, and the self-enhancing subscale has some long-winded, passive-voice clunkers, such as “It is my experience that thinking about some amusing aspect of a situation is often a very effective way of coping with problems.” In short, the HSQ as a whole has a reading level that is appropriate for adult populations, but it is problematic that the subscales vary in their readability. Future item revision should aim for consistent readability levels so that differences in subscale scores are not contaminated by factors related to reading comprehension and verbal abilities.

Another notable item feature is polarity—whether the item is phrased in a positive or negative direction. The virtues, problems, and consequences of reverse-scored items remain controversial [45,46,47,48], and there is clearly no single approach that is best for all tools, populations, and purposes. Nevertheless, if a scale includes reversed items, they should be distributed evenly across subscales. The four HSQ subscales vary notably in the proportion of reverse-coded items (see Table 2). For the affiliative subscale, more than half of the items (five of eight items) are reverse coded. The aggressive scale is similar, with half of the items (four of eight items) reversed. The self-enhancing and self-defeating subscales, however, each have only one reverse-coded item. The subscales are thus imbalanced, ranging from mostly to minimally reversed, and this imbalance is systematic: the two interpersonal subscales are at least 50% reversed, whereas the two intrapersonal subscales are only 12.5% reversed.

In addition, many of the reverse-coded items use simple negation, such as “don’t” and “not,” which is a widely criticized practice [48]. Respondents often miss these words and thus interpret the item in the wrong direction, which reduces internal consistency and increases item misfit [49]. Another consequence is reduced unidimensionality due to local dependence. Two similarly worded items that vary only in simple negation will often have a high residual correlation. This appeared for several HSQ items in the affiliative subscale, as we saw earlier. We do not have strong feelings about including or omitting reverse-scored items, but we see the imbalance in the proportion of reversed items across subscales and the predominance of simple negations as problematic for the HSQ, and it should be addressed in future item revisions.

## 4. Conclusions

In the present research, we conducted a psychometric analysis of the Humor Styles Questionnaire, one of the most widely used self-report scales in modern humor research. Because the scale is nearly 20 years old and has not yet been evaluated using Rasch and item response theory methods, a thorough evaluation can set the stage for future scale revision by highlighting strengths to preserve and opportunities for improvement. Based on our analyses, the HSQ has many strengths, so any future remodeling is more a matter of light renovations than of gutting the interior and starting over.

We should note some limitations of our analysis. First, our conclusions apply only to the English form of the HSQ. This scale has been widely translated into dozens of languages [6,8], often with noteworthy changes to the response scale and item wording to reflect distinct cultural inflections in the meanings and uses of humor [50]. Each translated form of the scale deserves its own separate analysis. Second, our primary analysis of the HSQ was grounded in the dataset that used a five-point response scale. Our findings provide evidence that the five-point scale has more consistent psychometric behavior and should be preferred to the traditional seven-point scale. Nevertheless, some of the item and scale parameters (e.g., difficulty, discrimination, and item fit) would be expected to differ slightly for the seven-point scale version.

We have focused our attention on psychometric aspects of the HSQ rather than on broader questions of its validity. Some researchers, however, have recently argued that the HSQ has questionable construct validity [51,52]. It is unclear, in our opinion, how much some of the methods used to challenge the scale’s validity (e.g., experimentally manipulating items) inform the complex issue of validity for the HSQ [53], especially in light of the large literature that suggests that it is a useful tool for humor inquiry. Validity is not a static feature of a scale but rather an evolving feature of the kinds of interpretations and claims that are legitimately afforded by a growing body of scholarship [54].

If researchers revise and refine the HSQ’s items, it would be wise to take advantage of the accumulated literature related to the validity of its underlying constructs, both favorable and critical. As one example, research since the HSQ was first published suggests that self-defeating humor probably has distinct sides: the maladaptive side that the HSQ intends to assess and an adaptive side reflecting healthy self-deprecation and humility [35,36]. The HSQ might not distinguish these as clearly as it could.

In conclusion, here are the major take-home messages from our analysis for the scale’s users and for researchers interested in refining and revising the scale. The HSQ has several strengths to preserve and build upon:

- The HSQ’s subscales are essentially unidimensional and yield scores with good reliability relative to their length.
- The items have essentially no gender-based differential item functioning, which is a striking finding for a scale with prominent gender differences. We see this as a major strength of the HSQ.
- Except for the affiliative subscale, all the subscales provide most of their information around the center of the trait, which is apt for measures of normal individual differences.

Based on our analyses, here are the major opportunities for improvement:

- The rating scale analysis indicates that seven points is probably too many. The five-point items were more consistently ordered, and a five-point scale is a sensible alternative. The behavior of a rating scale is an empirical question, so we would encourage future development of the scale to explore the behavior of alternative rating scales, such as four or six points.
- The affiliative subscale is simply too “easy” for the intended population, and it is much easier than the other subscales. We see the disparity in overall difficulty as a problem for the HSQ because overall subscale scores are not comparable. The varying difficulty levels are almost certainly an unintended consequence of the scale development process and something that should be targeted in future revisions. There is no ideal difficulty level, but scales that measure normal individual differences usually seek to distribute their items across the difficulty spectrum, thus allowing them to gain insight into a wide range of the trait.
- The items’ readability should be adjusted and harmonized. The subscales vary in sentence length and readability, which introduces unwanted variance.
- The proportion of reverse-scored items should be more similar across the subscales, and reversing with simple negation should be avoided.
- Some items are relatively low in discrimination and thus offer relatively little information. These are good candidates for item revision or deletion.
- According to local dependence statistics, a handful of items impair unidimensionality and are thus easy targets for item revision or deletion.

## Figures and Tables

**Table 1:** Humor styles questionnaire: Abbreviated item stems and psychometric features.

Item	Difficulty (*b*)	Slope (*a*)	Tau 1	Tau 2	Tau 3	Tau 4	Infit	Outfit	RMSD	Disordered	Local Dependence
Affiliative Style											
1. usually don’t laugh or joke around with others	−1.033	1.452	−1.204	−0.324	0.020	1.508	1.01	1.06	0.028		
5. don’t have to work hard at making other people laugh	−0.628	1.186	−1.447	−0.820	0.296	1.971	1.00	0.99	0.016		29
9. rarely make other people laugh by telling funny stories	−0.509	0.691	−1.198	−0.593	−0.446	2.237	1.01	1.06	0.038		
13. laugh and joke a lot with friends	−1.494	1.605	−1.052	−0.190	0.004	1.238	1.03	0.99	0.017		25
17. don’t like to tell jokes	−0.994	1.682	−1.014	−0.266	0.059	1.221	1.03	1.01	0.027		
21. enjoy making people laugh	−1.531	1.767	−0.784	−0.583	−0.069	1.436	1.02	0.99	0.015		
25. don’t often joke around with friends	−1.356	1.852	−0.863	−0.118	−0.164	1.144	1.07	1.01	0.024	Yes	13
29. can’t think of witty things to say with people	−0.690	0.809	−1.066	−0.472	−0.199	1.738	1.02	1.05	0.032		5
Self-Enhancing Style											
2. if feeling depressed, can cheer up with humor	−0.310	1.194	−1.764	−0.528	0.284	2.008	1.00	1.00	0.023		
6. when by myself, I’m amused by absurdities of life	−1.783	0.624	−1.704	−0.410	−0.006	2.120	1.01	0.99	0.032		30
10. if feeling upset I usually try to think of something funny to feel better	0.025	1.367	−1.490	−0.452	0.189	1.753	1.01	1.01	0.018		
14. my humorous outlook keeps me from getting overly upset	−0.300	1.156	−1.498	−0.330	0.246	1.583	1.01	1.02	0.021		
18. if by myself and unhappy, I think of something funny to cheer up	0.197	1.371	−1.601	−0.407	0.224	1.784	1.01	1.01	0.023		
22. if feeling sad, I lose my sense of humor	0.220	0.462	−2.921	0.055	0.223	2.643	1.00	1.02	0.037		
26. thinking about some amusing aspect of a situation is effective way of coping	−0.746	1.078	−1.376	−0.697	0.204	1.870	1.01	1.00	0.024		
30. can usually find things to laugh about when by myself	−1.586	0.527	−1.706	−0.432	−0.065	2.203	1.01	0.99	0.035		6
Aggressive Style											
3. if someone makes a mistake I will tease them	0.137	0.828	−1.801	−0.518	0.392	1.927	1.01	1.01	0.022		
7. people are never offended by my sense of humor	0.371	0.671	−2.602	−0.371	0.966	2.007	1.00	1.02	0.021		
11. when telling jokes I am not concerned about how other people are taking it	0.557	0.582	−2.333	0.433	−0.368	2.269	1.00	1.02	0.025	Yes	
15. do not like when people use humor for putting someone down	0.598	0.939	−1.108	−0.220	0.013	1.315	1.01	1.01	0.016		
19. sometimes I think of something funny that I can’t stop myself from saying it, even if not appropriate	−0.140	0.584	−1.942	−0.143	−0.245	2.330	1.01	1.01	0.024	Yes	
23. never participate in laughing at others	−0.237	0.655	−1.918	−0.612	0.317	2.213	1.00	1.01	0.026		
27. if I don’t like someone, I use humor to put them down	1.114	0.671	−1.300	0.274	−2.01	1.227	1.01	1.01	0.025	Yes	
31. if something is funny, I will not laugh if someone will be offended	−0.138	0.977	−1.572	−0.052	0.369	1.256	1.01	1.01	0.023		
Self-Defeating Style											
4. let people make fun at my expense	0.272	0.859	−2.076	−0.300	0.421	1.955	1.01	1.02	0.032		24
8. get carried away in putting myself down	0.455	1.859	−1.342	−0.235	0.186	1.392	1.01	1.00	0.016		
12. try to make people accept me by saying something funny about my faults	0.036	0.934	−1.699	−0.481	0.059	2.122	1.01	1.02	0.034		
16. don’t say funny things to put myself down	0.113	0.771	−1.933	−0.321	0.089	2.165	1.00	1.04	0.040		
20. go overboard in putting myself down when trying to be funny	0.785	1.973	−1.320	−0.225	0.174	1.371	1.02	0.97	0.015		
24. seem to be the one that other people joke about	1.016	0.536	−2.767	−0.128	0.290	2.605	1.01	1.02	0.033		4, 32
28. if unhappy, I cover it up by joking	−0.251	0.381	−1.747	−0.366	−0.472	2.584	1.00	1.01	0.029	Yes	
32. letting others laugh at me keeps my friends in good spirits	0.098	0.989	−1.402	−0.674	0.172	1.905	1.01	0.99	0.033		24

Note: The items are abbreviated item stems, not the complete wording. The item numbers are from the scale’s original article, where the complete items can also be found (Martin et al. [2], pp. 58, 59). The statistics are from the primary dataset with the 5-point response scale. Difficulty reflects how easy or hard it is to endorse an item: higher values correspond to lower endorsement rates: higher trait levels are needed to get higher scores on these items. Discrimination reflects how well items discriminate between different levels of the underlying trait. Items with higher discrimination values provide more information and can more reliably separate participants with similar ability levels. The Tau values are the four thresholds that represent the boundaries between the 5 response options. The Tau values represent the trait level, on the underlying trait scale, on which someone has a 50:50 chance of giving the lower vs. the higher response. Disordered indicates if the item’s thresholds are disordered. Local dependence shows which other items, if any, an item has a notable residual correlation with (i.e., a*Q*_3_ > |0.20|). RMSD = root mean squared deviation item-fit statistic. RMSD values greater than 0.02 and 0.05 reflect “small” and “medium” misfit, respectively.

**Table 2:** Humor Styles Questionnaire (HSQ) subscale features.

Subscale	Cronbach’s α	Omega (ω_H_)	EAP Reliability	Gender Difference (*d*)	Reversed Items	Flesch Reading Ease	Gunning Fog Index	Sentence Length (Words)
Affiliative	0.86	0.77	0.85	−0.19 [−0.31, −0.08]	5	71.0	6.7	11.13
Self-enhancing	0.82	0.68	0.84	−0.09 [−0.20, 0.02]	1	57.5	11.7	18.38
Aggressive	0.79	0.68	0.80	−0.48 [−0.60, −0.36]	4	67.3	9.2	17.25
Self-defeating	0.82	0.71	0.86	−0.18 [−0.30, −0.07]	1	70.1	10.2	19.75

Note: The findings are from the 5-point HSQ dataset. ω*_H_* = omega hierarchical. Gender is scored 0 = men, 1 = women, so negative Cohen’s *d* values reflect relatively higher scores for men; brackets contain the 95% confidence intervals around *d*. Text is more readable when it has higher Reading Ease scores and lower Fog Index scores. Sentence length is the average number of words per item.

## Supplementary Materials

The raw data and R files are publicly available at Open Science Framework (https://osf.io/7zy2h/). We invite researchers to reanalyze and reuse the data for their own purposes.

## References

[1] Turner M.J., Hesford J.W. (2019). The Impact of Renovation Capital Expenditure on Hotel Property Performance. Cornell Hosp. Q..

[2] Martin R.A., Puhlik-Doris P., Larsen G., Gray J., Weir K. (2003). Individual Differences in Uses of Humor and Their Relation to Psychological Well-Being: Development of the Humor Styles Questionnaire. J. Res. Personal..

[3] Kuiper N.A., Zeigler-Hill V., Shackelford T. (2016). Model of humor styles. Encyclopedia of Personality and Individual Differences.

[4] Bond T.G., Yan Z., Heine M. (2020). Applying the Rasch Model: Fundamental Measurement in the Human Sciences.

[5] DeMars C. (2010). Item Response Theory.

[6] Kuiper N.A., Zeigler-Hill V., Shackelford T. (2020). Humor Styles Questionnaire. Encyclopedia of Personality and Individual Differences.

[7] Martin R.A., Ford T. (2018). The Psychology of Humor: An Integrative Approach.

[8] Schermer J.A., Rogoza R., Kwiatkowska M.M., Kowalski C.M., Aquino S., Ardi R., Bolló H., Branković M., Chegeni R., Crusius J. (2019). Humor Styles across 28 Countries. Curr. Psychol..

[9] Scheel T., Gerdenitsch C., Korunka C. (2016). Humor at Work: Validation of the Short Work-Related Humor Styles Questionnaire (SwHSQ). Humor.

[10] Anlı G. (2019). Reliability and validity studies of the turkish version of humor styles questionnaire for children. Curr. Psychol..

[11] Fox C.L., Dean S., Lyford K. (2013). Development of a humor styles questionnaire for children. Humor.

[12] Hofmann J., Platt T., Lau C., Torres-Marín J. (2020). Gender Differences in Humor-Related Traits, Humor Appreciation, Production, Comprehension, (Neural) Responses, Use, and Correlates: A Systematic Review. Curr. Psychol..

[13] Mendiburo-Seguel A., Páez D., Martínez-Sánchez F. (2015). Humor Styles and Personality: A Meta-analysis of the Relation between Humor Styles and the Big Five Personality Traits. Scand. J. Psychol..

[14] Plessen C.Y., Franken F.R., Ster C., Schmid R.R., Wolfmayr C., Mayer A.M., Maierwieser R.J. (2020). Humor Styles and Personality: A Systematic Review and Meta-Analysis on the Relations between Humor Styles and the Big Five Personality Traits. Personal. Individ. Differ..

[15] Schneider M., Voracek M., Tran U.S. (2018). “A Joke a Day Keeps the Doctor Away?” Meta-Analytical Evidence of Differential Associations of Habitual Humor Styles with Mental Health. Scand. J. Psychol..

[16] Greengross G., Silvia P.J., Nusbaum E.C. (2020). Sex Differences in Humor Production Ability: A Meta-Analysis. J. Res. Personal..

[17] Greengross G. (2020). Sex and Gender Differences in Humor: Introduction and Overview. Humor.

[18] Yaprak P., Güçlü M., Ayyildiz Durhan T. (2018). The Happiness, Hardiness, and Humor Styles of Students with a Bachelor’s Degree in Sport Sciences. Behav. Sci..

[19] Markos A., D’Enza A.I., van de Velden M. (2019). Beyond Tandem Analysis: Joint Dimension Reduction and Clustering in R. J. Stat. Softw..

[20] Yentes R.D., Wilhelm F. (2018). Careless: Procedures for Computing Indices of Careless Responding, R Package, Version 1.1.3. https://cran.r-project.org/package=careless.

[21] (2020). R: A Language and Environment for Statistical Computing. https://www.R-project.org.

[22] Revelle W. (2020). Psych: Procedures for Psychological, Psychometric, and Personality Research, R Package Version 2.0.9. https://CRAN.R-project.org/package=psych.

[23] Robitzsch A., Kiefer T., Wu M. (2020). TAM: Test Analysis Modules, R Package Version 3.6-8. https://CRAN.R-project.org/package=TAM.

[24] Dijkstra P., Barelds D., Ronner S., Nauta A. (2011). Humor Styles and Their Relationship to Well-Being among the Gifted. Gift. Talent. Int..

[25] Markey P.M., Suzuki T., Marino D.P. (2014). The Interpersonal Meaning of Humor Styles. Humor.

[26] MacDonald K.B., Kumar A., Schermer J.A. (2020). No Laughing Matter: How Humor Styles Relate to Feelings of Loneliness and Not Mattering. Behav. Sci..

[27] Sirigatti S., Penzo I., Giannetti E., Stefanile C. (2014). The Humor Styles Questionnaire in Italy: Psychometric Properties and Relationships with Psychological Well-Being. Eur. J. Psychol..

[28] Linacre J.M. (2002). Optimizing Rating Scale Category Effectiveness. J. Appl. Meas..

[29] Slocum-Gori S.L., Zumbo B.D. (2011). Assessing the Unidimensionality of Psychological Scales: Using Multiple Criteria from Factor Analysis. Soc. Indic. Res..

[30] Hayton J.C., Allen D.G., Scarpello V. (2004). Factor Retention Decisions in Exploratory Factor Analysis: A Tutorial on Parallel Analysis. Organ. Res. Methods.

[31] Velicer W. (1976). Determining the Number of Components from the Matrix of Partial Correlations. Psychometrika.

[32] Marais I., Christensen K.B., Kreiner S., Mesbah M. (2013). Local Dependence. Rasch Models in Health.

[33] Christensen K.B., Makransky G., Horton M. (2017). Critical Values for Yen’s Q3: Identification of Local Dependence in the Rasch Model Using Residual Correlations. Appl. Psychol. Meas..

[34] Chen W.H., Thissen D. (1997). Local Dependence Indices for Item Pairs Using Item Response Theory. J. Educ. Behav. Stat..

[35] Heintz S., Ruch W. (2018). Can Self-Defeating Humor Make You Happy? Cognitive Interviews Reveal the Adaptive Side of the Self-Defeating Humor Style. Humor.

[36] Tsukawaki R., Imura T. (2020). The Light and Dark Side of Self-Directed Humor: The Development and Initial Validation of the Dual Self-Directed Humor Scale (DSDHS). Personal. Individ. Differ..

[37] Köhler C., Robitzsch A., Hartig J. (2020). A Bias-Corrected RMSD Item Fit Statistic: An Evaluation and Comparison to Alternatives. J. Educ. Behav. Stat..

[38] Buchholz J., Hartig J. (2019). Comparing Attitudes across Groups: An IRT-Based Item-Fit Statistic for the Analysis of Measurement Invariance. Appl. Psychol. Meas..

[39] Ostini R., Nering M.L. (2006). Polytomous Item Response Theory Models.

[40] Choi S.W., Gibbons L.E., Crane P.K. (2011). Lordif: An R Package for Detecting Differential Item Functioning Using Iterative Hybrid Ordinal Logistic Regression/Item Response Theory and Monte Carlo Simulations. J. Stat. Softw..

[41] Choi S.W., Gibbons L.E., Crane P.K. (2016). Lordif: Logistic Ordinal regression Differential Item Functioning Using IRT, R package version 0.3.3. https://cran.r-project.org/package=lordif.

[42] Menard S. (2000). Coefficients of Determination for Multiple Logistic Regression Analysis. Am. Stat..

[43] Meade A.W. (2010). A Taxonomy of Effect Size Measures for the Differential Functioning of Items and Scales. J. Appl. Psychol..

[44] DuBay W.H. (2004). The Principles of Readability.

[45] Conrad K.J., Wright B.D., McKnight P., McFall M., Fontana A., Rosenheck R. (2004). Comparing Traditional and Rasch Analyses of the Mississippi PTSD Scale: Revealing Limitations of Reverse-Scored Items. J. Appl. Meas..

[46] Netemeyer R.G., Bearden W.O., Sharma S. (2003). Scaling Procedures: Issues and Applications.

[47] Primi R., De Fruyt F., Santos D., Antonoplis S., John O.P. (2020). True or False? Keying Direction and Acquiescence Influence the Validity of Socio-Emotional Skills Items in Predicting High School Achievement. Int. J. Test..

[48] Suárez-Alvarez J., Pedrosa I., Lozano Fernández L.M., García-Cueto E., Cuesta M., Muñiz J. (2018). Using Reversed Items in Likert Scales: A Questionable Practice. Psicothema.

[49] Woods C.M. (2006). Careless Responding to Reverse-Worded Items: Implications for Confirmatory Factor Analysis. J. Psychopathol. Behav. Assess..

[50] Jiang T., Li H., Hou Y. (2019). Cultural Differences in Humor Perception, Usage, and Implications. Front. Psychol..

[51] Heintz S., Ruch W. (2015). An Examination of the Convergence between the Conceptualization and the Measurement of Humor Styles: A Study of the Construct Validity of the Humor Styles Questionnaire. Humor.

[52] Ruch W., Heintz S. (2017). Experimentally Manipulating Items Informs on the (Limited) Construct and Criterion Validity of the Humor Styles Questionnaire. Front. Psychol..

[53] Martin R.A. (2015). On the Challenges of Measuring Humor Styles: Response to Heintz and Ruch. Humor.

[54] Messick S. (1995). Validity of Psychological Assessment: Validation of Inferences from Persons’ Responses and Performances as Scientific Inquiry into Score Meaning. Am. Psychol..

